# Forensic Age Estimation of Chinese Malaysian Adults by Evaluating Occlusal Tooth Wear Using Modified Kim's Index

**DOI:** 10.1155/2017/4265753

**Published:** 2017-10-10

**Authors:** Chai Kit Lu, Margaret Chia Soo Yee, Spoorthi Banavar Ravi, Rohit Pandurangappa

**Affiliations:** School of Dentistry, International Medical University, 126/Jalan 19/155B, Bukit Jalil, 57000 Kuala Lumpur, Malaysia

## Abstract

**Background and Objective:**

Evaluation of dental attrition is an easy and relatively accurate approach to estimating the age of an adult either ante- or postmortem for some specific population. Dental attrition represents a progressive physiological age change that can be measured using variety of indices to aid as an adjunct in forensic age estimation. Some of the previously proposed indices have their own practical limitations. This paper focuses on using modified Kim's criteria to score dental attrition to estimate the age of Chinese Malaysian adults and validate it.

**Methodology:**

Tooth wear was evaluated on 190 dental models of Chinese Malaysian adults (age range: 20–60 years) using modified Kim's index to custom-derive a population specific linear equation. The same equation was validated further on new 60 dental casts.

**Results and Conclusion:**

Regression analysis revealed good correlation between age and teeth wear and lower standard error of estimate. Test of regression on a test sample (*n* = 30 pairs, age range: 20–60 years) showed insignificant difference between predicted versus the actual age with statistically acceptable mean absolute difference. These data suggest that modified Kim's index can be used effectively in forensic age estimation.

## 1. Introduction

Establishing one's identity is an important aspect in any forensic casework. Numerous contributing factors like age, sex, population designation, and stature prediction help in reconstructing the identity. Among these factors, age estimation plays a vital role in such scenarios. This is especially true when the police or the investigators already have a putative age; a forensic age estimation that is close to the presumed age provides clarity in the line of investigation [1]. Since Gustafson's scoring system [2] for estimation of chronological age from human teeth, there have been several reports on improved methodologies [3–11] including digital techniques in this field [1]. However, some of these methods require tooth extraction and preparation of microscopic sections of teeth. Such invasive methods are not applicable to estimating the chronological age of a living person. Other noninvasive methods using radiographs wherein age is being predicted based on dental maturation [12] or the ratio between pulp chamber and the tooth have also been reported [13]. Kim et al., in the year 2000, originally proposed a system to objectively score the wear of the teeth of an adult individual [14]. Researchers [15, 16] have reported using this scoring system to estimate the age of an individual. Practical requirements such as presence of entire set of sound and healthy dentition for the system limit its usefulness. To overcome this, a proposed modification of the original Kim's system was used to assess its practical applicability, on a Chinese Malaysian population.

The aim of our study was to evaluate the validity of modified Kim's index as a practical clinical method of recording the degree of occlusal wear and estimating the chronological age.

## 2. Materials and Methods

The study was based on 250 randomly selected {125 pairs (95 pairs for the reference group and 30 pairs for the test group)} maxillary and mandibular full arch casts obtained from heterogeneous samples of males and females (53 males, 72 females) belonging to Chinese Malaysian origin, ranging in age from 20 to 60 years. The study samples were further divided into four groups (20–30 years, 31–40 years, 41–50 years, and 51–60 years). Age and gender distributions are presented in Table 1. While randomly selecting the casts for the study, individuals having severe malocclusion that could affect the occlusal wear and edentulous casts were excluded from the study.

Only the posterior teeth (excluding third molars) were considered for tooth wear evaluation. Tooth wear score was categorised using 0–10 point scale based on the amount and pattern of tooth wear on the occlusal surface following the modified Kim's index, as given in Table 2. The full arch casts were evaluated by 2 examiners under regular room light using a magnifying glass. Two examiners scored the occlusal wear individually after a mutual calibration session. Twenty pairs of randomly selected casts were used to calibrate and evaluate the concordance between intraexaminer and interexaminer scoring method.

A total of 190 (95 pairs) full arch casts were evaluated first. The subjects were categorised into four groups (group 1 to group 4) based on their age as 21–30 years, 31–40 years, 41–50 years, and 51–60 years, respectively. All the casts were coded before their scoring to avoid any type of bias. After scoring the occlusal wear in all the 95 pairs of full arch casts, the casts were decoded to know the actual age and the gender. This sample of 95 pairs was referred to as “reference data set.” The data generated from this group was used to derive a statistical linear regression equation. Further, the same equation was applied on later collected cases called “test data set” (*n* = 30 pairs) to predict the age of the subjects.

The difference between the actual age and the estimated age was calculated for every case in both the sets and the mean absolute difference (MAD) was calculated. This MAD (irrespective of the positive or the negative value) would represent the average magnitude of difference that was used as an average measure of accuracy. The differences in age prediction were calculated as to lie within ±3-, ±5-, and ±10-year range of the actual age, along with errors that are ≤10 years and ≥15 years as per Solheim and Sundnes [17] who have classified the former as “acceptable” and the latter as “unsatisfactory” in forensic age estimation.

### 2.1. Statistical Analysis

For all statistical evaluations, SPSS 2015 for windows was used (SPSS Inc., Chicago, IL: currently IBM corporation, New York). The statistical significance for all the tests were set at 0.05. To evaluate inter- and intraexaminer variability, interclass correlation coefficient (ICC) was employed. Gender difference in occlusal tooth wear scores between each tooth was evaluated using independent *t*-test. Correlation between occlusal tooth wear scores and the actual age was established using Karl Pearson correlation test. The regression analysis using the data from the reference data set provided a linear equation that when used would predict the age of the individual belonging to the test set based on the independent variables observed. In our study the age was the dependant variable and the teeth wear scores from 16 teeth (all premolars and molars) were independent variables. The equation derived was based on the statistical regression formula, *y* = *a* + *b*(*x*). Multivariate regression analysis was followed as we had multiple independent variables.

(1) Y=a+b1x14+b2x15+b3x16+b4x17+b5x24+b6x25+b7X26+b8x27+b9x34+b10x35+b11x36+b12x37+b13x44+b14x45+b15x46+b16x47.

## 3. Results

**Figure 1:**
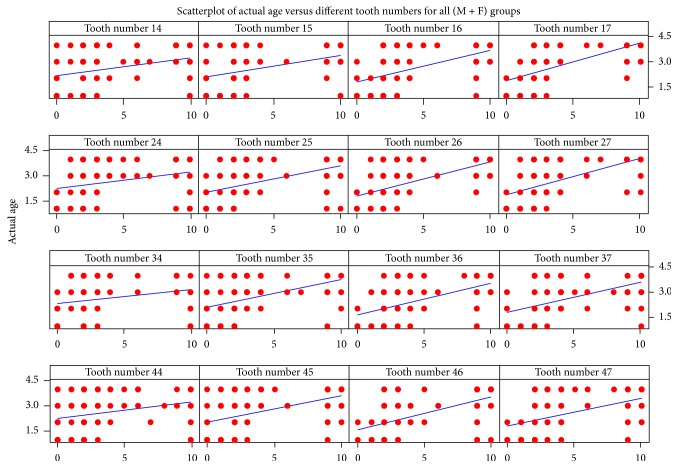
Scatter plot showing correlation of actual age with occlusal wear scores.

The ICC scores of 0.97 (*P* = 0.02) and 0.95 (*P* = 0.03) showed very good concordance between the examiners. The mean occlusal wear scores of all teeth in the reference group were marginally higher in females than in males, but this difference was not statistically significant (3.62 versus 3.19, *P* > 0.005, Table 3). The mean wear scores of molars were significantly higher than the mean wear scores of premolars (3.88 versus 3.31, *P* = 0.036). Comparison of the mean occlusal wear scores among four different age groups in the reference data set group was statistically significant for all the teeth except teeth 24, 34, and 44. The mean wear scores of all the teeth across different age group showed a gradual increase with the age (Table 4). The correlation test revealed positive correlation between occlusal tooth wear scores and age with coefficient of determination (*r*^2^) of 0.82 for males and 0.74 for females (Table 5). The standard errors* of age* were 7.37 and 7.26 for males and females, respectively. The degree of correlation was moderately positive between the tooth wear scores of all the teeth examined. The correlation coefficient values of all the molar teeth were higher than the premolars; the values were between 0.272 and 0.568 (Table 6, Figure 1).

Based on the regression analysis, a specific equation for age estimation was derived. This was based on *y* = *a* + *b*(*x*), where the intercept (*a*) and the regression coefficient (*b*) for each tooth were derived as shown in Table 7. The complete equation derived for predicting the age was

(2) Predicted  Age=20.6+0.01914+0.63515+0.08716+0.83817+0.36824+0.01625+0.56726+0.88927−0.66534+0.17235+0.30536+0.82737+0.36144+0.11245+0.58946+0.09747.

Further, by scoring all 16 teeth (independent variables: 14, 15, 16, 17, 24, 25, 26, 27, 34, 35, 36, 37, 44, 45, 46, and 47) the values were substituted in the equation to predict the age of an individual belonging to “test data set group.” The accuracy of age prediction using the modified Kim's index was assessed by calculating the MAD as shown in Table 8. Predicted ages were within ±3 years and ±5 years of the actual age in 20% and 40% and 13.33 and 33.33% of males and females, respectively. Similarly, the number of subjects whose predicated age ranged within ±10 years was 66.66% for both males and females. Thirty-three percent of males and females had a prediction of their age to be >10 years of difference (Table 8). The mean absolute difference that truly represents the accuracy of the formula between the actual age and the predicted age was marginally higher in females than males, but it was not statistically significant.  Table 9 shows the complete data and the age prediction of all the samples included in the reference group as well as the test group.

## 4. Discussion

Among all the types of regressive alterations affecting the teeth, attrition is the only change considered to be physiological that progressively increases with the advancing age. The rest are considered to be due to pathological process. Predicting the age of an individual based only on occlusal wear may not be an accurate estimate as the occlusal wear may be dependent on many factors [18] like dietary habits, mastication, pressure transmitted during mastication, number of teeth present, presence or absence of opposing teeth, presence of artificial teeth, geographic and environmental factors, parafunctional habits like bruxism, and factors like malocclusion. The relation between occlusal wear and aging was evaluated among indigenous Amazon population and suggested that tooth wear is a poor estimator of chronological age in the urban population; however it has a strong association with age for more remote indigenous populations [19]. However, some rare scenarios demand the forensic team to predict the age with limited available information. This may be due to cases like unknown illegal immigration, cases not permitting undertaking invasive samples, and so on. Predicting the age of an individual in such cases based on noninvasive methods like scoring occlusal wear can be an invaluable adjunct for the forensic team.

Numerous systems or indices are available for evaluating the occlusal wear till date [14, 20–23]; however there is no universally accepted method. Kim et al. in the year 2000 presented their 0–8 point method of scoring or recording the occlusal wear and it was shown that their new system was a reliable and accurate method for age estimation [14]. Though Kim's system aims at evaluating the degree of occlusal wear, the system mandatorily requires sound and healthy teeth to be evenly considered. This becomes the biggest practical drawback as oral conditions like dental caries, fractures, and missing teeth are omnipresent in most populations worldwide. Clinicians would always prefer to have a system that is more versatile and that can be applied in widest of the conditions possible. To address this issue, Yun et al. [15] proposed a modification to the original Kim's index, where they included 2 additional points to the already existing 0–8 point scale (Table 2). The proposed modification had 0–10 point scale in evaluating the degree of occlusal wear. In the modified system, all of teeth except the 3rd molars are included and the system also scores for unsound, restored, and even the missing teeth. The basis for such additional points in their modification system is the observed good correlation between such oral conditions and aging. This has been validated on Korean population by a study conducted by Yun et al. [15]. The study by Vieira et al. [19] on indigenous Amazon population differs by saying that the results are population specific which is basically dependent upon their dietary habits. Hence our study aimed at evaluating the clinical validity of modified Kim's index in predicting the age of Chinese Malaysian population. More importantly, we modified the way the index was used. Instead of considering all the teeth excluding third molars, we considered only the posterior teeth. This was based on the fact that posterior teeth are more prone for occlusal wear rather than the anterior teeth. Posterior teeth have a wider occlusal table making them more vulnerable than the anterior teeth. Previous studies [15, 16] report significantly higher wear among molars and premolars than the incisors and canines. Moreover, this approach will be practically more applicable when compared to using the original Kim's index for forensic applications.

In the present study, 250 (125 pairs) full arch casts were evaluated for occlusal wear. The degree of correlation was moderately positive between the tooth wear scores of all the teeth examined with the correlation coefficient values ranging from 0.272 and 0.568. This could be attributed to the fact that occlusal wear can have other confounding factors as explained by Johansson et al. [18], but nevertheless, the positive relation between aging and teeth wear scores is observed in our study as shown in the scatter plot diagrams. The results showed that the amount of occlusal wear was marginally higher in females than in males; this is in contrast to studies done by many researchers [15, 16]; however this difference was not statistically significant. This is in contrast to previous studies that report significantly higher attrition rates in males than in females [16]. This could be attributable to the dietary habits of the population studied. Our observations indicate similar dietary habits between the males and the females belonging to Chinese Malaysian population. Similarly higher degree of occlusal attrition was noted in older age group than the younger age groups. This is in accordance with previous studies [15, 16, 24, 25]. The mean wear scores of molars were found to be more than the mean occlusal wear scores of the premolars. This can be due to the fact that the molars bear more masticatory load than the premolars. The other reason for this could be due to the eruption sequence. First molars erupt significantly much earlier in the oral cavity than the premolars and hence are exposed earlier and for a longer duration to the physiological wear. This finding of our study is consistent with the previous reports [15, 16, 26–28]. Telang et al. conducted a study on a population of 120 Indians using the Kim's index [16]. The study predicted the age to be ±5 years in 70% and 68.3% and 50% and 50.1% to be ±3 years in males and females, respectively. These results are similar to the findings of our study. Similarly the findings of study done by Yun et al. on sample of 1092 randomly selected pairs also showed similar results [15]. No study previously described in the literature has evaluated occlusal wear and derived a population specific formula using regression analysis and had tested the same formula on a control “test data set group.” In our study, we tested the derived formula on further collected cases called “test data set.” The Mean absolute difference (MAD) between the predicted age and the actual age was within the acceptable range as described by Solheim and Sundnes [17] in 66.66% in both males and females. The purpose of such an approach was to estimate the accuracy and practical applicability of the population specific formula in such forensic case work and not halt at an interpretation based correlation between teeth wear scores and the actual age. Moreover, Gorard [29] indicates that statistical estimates based on the standard error of estimate may give misleading interpretations and mentions that MAD is more appropriate in such cases and practical applications. When the predicted ages were compared with the actual age the difference between them was not statistically significant. The limitations of our study include limited number of sample of 250 pairs; a larger sample size might give a better regression formula with lower error rates.

In conclusion, we propose a method to evaluate occlusal wear using a modified index and this was shown to give a good age estimate even when tried on the test sample. These data suggest that the modified Kim's index can be used as an adjunct to quantify occlusal wear to predict the age of an adult individual. We do believe that there is still no universally acceptable method for evaluating occlusal wear; however the method of modifying the already existing index to be tailored to practical applicability renders it more useful than the original Kim's index.

## Figures and Tables

**Table 1:** Sample distribution across gender and different age group in the reference group.

Reference data set	*n*	Gender	
		M	F
Age group			
20–30	29	14	15
31–40	18	07	11
41–50	24	08	16
51–60	24	10	14
Total	*95*	*38*	*57*

Test data set	*30*	*15*	*15*

Total	*125*	*53*	*72*

**Table 2:** Modified Kim's index to score the teeth wear [14].

Score	Premolar	Molar
(0)	No visible wear	
(1)	1P/1L	1P/1L/2P/2L
(2)	2P/2L/1S/1B	3P/3L/4P/4L/1S/1B/2S/2B
(3)	2S/2B	3S/3B/4S/4B
(4)	Wear on more than 2/3 of occlusal surfaces	
(5)	1Pc/1Lc	1Pc/1Lc/2Pc/2Lc
(6)	2Pc/2Lc/1Sc/1Bc	3Pc/3Lc/4Pc/4Lc/1Sc/1Bc/2Sc/2Bc
(7)	2Sc/2Bc	3Sc/3Bc/4Sc/4Bc
(8)	Concavity on more than 2/3 of occlusal surfaces	
(9)	Filling, ^*∗*^caries, ^*∗*^crown (all teeth)	
(10)	Missing, stump of tooth, pontic, denture (all teeth)	

^*∗*^If the extent of the filling materials or caries does not exceed 1/3 of the occlusal surface so that the degree of occlusal wear can be determined, the pertinent score should be given; P, point like wear facet less than ca. 1 mm in diameter; L, linear wear facet less than ca. 1 mm in width; S, surface like wear facet greater than ca. 1 mm in diameter; B, band like wear facet greater than ca. 1 mm in width or wear facet involving more than two surface like wear facets; “c” (concavity), the wear of dentin; in the situation where a tooth has several different degrees of occlusal wear, the highest degree should be selected as the occlusal wear score.

**Table 3:** Comparison of the mean occlusal wear scores of each tooth between males and females using independent *t*-test.

Tooth	Gender	*N*	Mean	Std. deviation	*P* value
14	Male	38	3.24	2.94	0.400
	Female	57	3.81	3.39	
15	Male	38	3.55	3.52	0.890
	Female	57	3.46	3.17	
16	Male	38	3.18	2.81	0.117
	Female	57	4.23	3.35	
17	Male	38	2.32	2.16	0.098
	Female	57	3.19	2.72	
24	Male	38	3.03	2.85	0.698
	Female	57	3.26	2.94	
25	Male	38	2.92	3.17	0.324
	Female	57	3.58	3.17	
26	Male	38	3.50	3.15	0.661
	Female	57	3.79	3.14	
27	Male	38	2.79	2.57	0.575
	Female	57	3.09	2.51	
34	Male	38	2.53	2.90	0.517
	Female	57	2.93	3.01	
35	Male	38	2.29	2.58	0.639
	Female	57	2.53	2.28	
36	Male	38	4.42	3.39	0.701
	Female	57	4.70	3.54	
37	Male	38	3.53	3.25	0.601
	Female	57	3.86	2.88	
44	Male	38	2.58	2.82	0.393
	Female	57	3.12	3.16	
45	Male	38	2.79	2.97	0.472
	Female	57	3.23	2.85	
46	Male	38	4.45	3.34	0.477
	Female	57	4.96	3.54	
47	Male	38	3.95	3.24	0.601
	Female	57	4.30	3.16	

**Table 4:** Comparison of mean wear scores of each tooth across different age groups using ANOVA.

Tooth number	Age								ANOVA test *F* value	*P* value
	21–30		31–40		41–50		51–60			
	Mean	SD	Mean	SD	Mean	SD	Mean	SD		
14	2.17	2.82	3.29	2.78	4.38	3.17	4.75	3.43	3.839	0.012
15	1.50	1.85	2.47	1.86	5.58	3.55	4.63	3.61	11.065	0.000
16	1.77	1.61	2.65	2.46	5.50	3.26	5.50	3.23	13.089	0.000
17	1.03	0.96	2.41	1.78	3.50	2.52	4.75	2.79	14.960	0.000
24	2.37	3.18	2.76	2.74	3.58	2.34	4.04	2.93	1.817	0.150
25	1.37	1.88	2.88	2.48	4.92	3.60	4.46	3.15	8.696	0.000
26	1.57	0.86	2.88	2.49	4.83	3.23	5.71	3.51	12.946	0.000
27	1.30	0.92	3.06	2.54	3.29	1.94	4.67	3.14	10.626	0.000
34	1.73	2.96	2.65	2.87	3.50	2.93	3.42	2.75	2.192	0.094
35	1.00	0.87	2.29	2.22	3.29	2.46	3.46	2.89	7.321	0.000
36	2.30	2.45	3.53	2.76	5.75	3.27	7.04	3.14	13.798	0.000
37	1.50	0.82	3.41	2.68	5.29	3.11	5.17	3.24	13.095	0.000
44	1.83	2.91	2.71	2.82	3.38	2.93	3.92	3.06	2.479	0.066
45	1.17	0.91	3.24	2.99	3.96	2.84	4.38	3.37	8.294	0.000
46	2.87	2.61	3.12	2.38	5.21	3.12	7.83	3.09	15.852	0.000
47	2.13	2.08	3.59	3.11	5.63	3.12	5.63	2.99	9.809	0.000

**Table 5:** Karl Pearson's correlation coefficient (*R*), coefficient of determination (*R*^2^), and standard error of the estimates for the collected data.

	Correlation coefficient (*R*)	*R* square	Std. error of the estimate
All (M & F)	0.806	0.649	8.176
Male (M)	0.908	0.824	7.377
Female (F)	0.864	0.747	7.264

**Table 6:** Correlation between actual age and tooth wear scores by Karl Pearson's correlation.

Tooth number	Correlation between age and tooth wear scores	
	Pearson correlation	*P* value
14	0.352	0.000
15	0.464	0.000
16	0.551	0.000
17	0.568	0.000
24	0.276	0.007
25	0.445	0.000
26	0.563	0.000
27	0.507	0.000
34	0.272	0.008
35	0.448	0.000
36	0.529	0.000
37	0.562	0.000
44	0.282	0.006
45	0.467	0.000
46	0.558	0.000
47	0.498	0.000

**Table 7:** The intercept and correlation coefficient (*β* coefficient) observed for multiple regression.

Constant (intercept)	20.6
*β* coefficient for each tooth	
14	0.019
15	0.635
16	0.087
17	0.838
24	0.368
25	0.016
26	0.567
27	0.889
34	0.665
35	0.172
36	0.305
37	0.827
44	0.361
45	0.112
46	0.589
47	0.097

**Table 8:** Comparison of accuracy of linear equation in predicting the age of the individuals of the test group.

Group	Mean wear scores	Predicted age that lies within the actual age range (in years)				MAD (in years)
*Reference group *		*±3*	*±5*	*±10*	*>10*	
Male (*n* = 38)	3.18	13.15%	28.93%	71.03%	26.31%	4.50
		5/38	11/38	27/38	10/38	
Female (*n* = 57)	3.62	21.05%	29.82%	70.17%	29.82%	8.10
		12/57	17/57	40/57	17/57	
*Test group*						
Male (*n* = 15)	4.67	20%	40%	66.66%	33.33%	8.14
		2/15	6/15	10/15	5/15	
Female (*n* = 15)	4.22	13.33%	33.33%	66.66%	33.33%	8.67
		2/15	5/15	10/15	5/15	

**Table 9:** Master chart of the details regarding the data of the samples included for the study.

Subject number	Gender	Age group^*∗*^	Actual age	Estimated age (Y)	Age difference	Groups^*∗∗*^
(1)	F	1	24	27.664	3.664	2
(2)	F	1	23	33.887	10.887	4
(3)	F	3	50	52.215	2.215	1
(4)	M	3	41	46.115	5.115	3
(5)	M	1	25	33.018	8.018	3
(6)	F	1	24	30.552	6.552	3
(7)	F	1	24	30.455	6.455	3
(8)	M	1	23	37.889	14.889	4
(9)	M	1	23	30.033	7.033	3
(10)	M	2	33	34.775	1.775	1
(11)	F	2	35	26.546	−8.454	3
(12)	F	1	23	29.806	6.806	3
(13)	M	1	24	27.127	3.127	2
(14)	F	1	25	32.298	7.298	3
(15)	M	3	45	37.711	−7.289	3
(16)	M	1	22	30.326	8.326	3
(17)	M	3	49	42.981	−6.019	3
(18)	F	4	51	61.358	10.358	4
(19)	M	1	26	32.521	6.521	3
(20)	M	1	26	28.475	2.475	1
(21)	F	1	23	31.701	8.701	3
(22)	F	1	23	25.318	2.318	1
(23)	M	1	25	29.128	4.128	2
(24)	F	1	24	25.169	1.169	1
(25)	F	1	23	38.166	15.166	4
(26)	M	1	25	28.227	3.227	2
(27)	M	1	27	34.839	7.839	3
(28)	M	1	24	26.112	2.112	1
(29)	F	3	42	43.741	1.741	1
(30)	M	4	51	39.34	−11.66	4
(31)	F	1	23	27.22	4.22	2
(32)	F	3	47	54.247	7.247	3
(33)	F	1	21	48.486	27.486	4
(34)	M	3	46	49.175	3.175	2
(35)	F	1	27	44.471	17.471	4
(36)	M	2	39	45.959	6.959	3
(37)	F	1	24	34.068	10.068	4
(38)	F	4	57	82.351	25.351	4
(39)	F	4	51	42.457	−8.543	3
(40)	F	1	24	44.376	20.376	4
(41)	M	1	24	33.738	9.738	3
(42)	F	3	43	48.793	5.793	3
(43)	F	2	39	55.874	16.874	4
(44)	F	4	57	56.565	−0.435	1
(45)	M	1	23	25.135	2.135	1
(46)	F	3	43	53.165	10.165	4
(47)	M	1	23	28.464	5.464	3
(48)	F	4	60	48.014	−11.986	4
(49)	M	2	33	36.59	3.59	2
(50)	M	4	55	53.838	−1.162	1
(51)	F	4	51	42.153	−8.847	3
(52)	M	1	24	32.671	8.671	3
(53)	M	4	57	48.133	−8.867	3
(54)	F	3	48	52.35	4.35	1
(55)	M	4	54	36.281	−17.719	4
(56)	M	3	43	55.636	12.636	4
(57)	F	2	40	44.089	4.089	2
(58)	M	3	42	58.93	16.93	4
(59)	F	3	44	48.023	4.023	2
(60)	M	4	60	45.443	−14.557	4
(61)	F	3	45	39.854	−5.146	3
(62)	F	2	35	43.245	8.245	3
(63)	M	4	57	51.838	−5.162	3
(64)	F	4	52	61.7	9.7	3
(65)	F	3	48	38.479	−9.521	3
(66)	M	4	51	47.52	−3.48	2
(67)	F	2	33	27.558	−5.442	3
(68)	F	2	32	26.427	−5.573	3
(69)	F	4	55	57.428	2.428	1
(70)	M	4	53	78.387	25.387	4
(71)	F	2	31	31.836	0.836	1
(72)	F	4	51	51.94	0.94	1
(73)	F	2	40	52.74	12.74	4
(74)	F	4	59	55.329	−3.671	3
(75)	F	3	50	62.75	12.75	4
(76)	F	2	38	48.489	10.489	4
(77)	F	2	32	35.668	3.668	2
(78)	F	2	38	39.207	1.207	1
(79)	F	2	35	49.73	14.73	4
(80)	F	3	50	59.302	9.302	3
(81)	F	3	42	52.317	10.317	4
(82)	M	4	56	61.646	5.646	3
(83)	F	2	33	39.608	6.608	3
(84)	F	3	47	45.104	−1.896	1
(85)	F	4	59	50.995	−8.005	3
(86)	F	4	57	66.936	9.936	3
(87)	M	3	44	63.697	19.697	4
(88)	F	4	57	62.015	5.015	3
(89)	M	2	39	53.772	14.772	4
(90)	M	2	34	44.133	10.133	4
(91)	F	4	52	52.702	0.702	1
(92)	F	3	46	51.89	5.89	3
(93)	F	3	43	61.303	18.303	4
(94)	M	4	60	71.593	11.593	4
(95)	M	3	49	56.861	7.861	3
(96)	F	2	36	37.881	1.881	1
(97)	F	4	60	44.366	−15.634	4
(98)	M	3	48	40.623	−7.377	3
(99)	M	4	56	47.26	−8.74	3
(100)	F	2	36	40.501	4.501	2
(101)	M	3	47	47.054	0.054	1
(102)	M	4	58	37.511	−20.489	4
(103)	M	3	43	35.717	−7.283	3
(104)	M	4	57	47.256	−9.744	3
(105)	M	4	56	43.639	−12.361	4
(106)	M	4	52	41.936	−10.064	4
(107)	M	2	34	38.625	4.625	2
(108)	M	3	48	43.957	−4.043	2
(109)	M	3	46	42.507	−3.493	2
(110)	M	2	32	33.688	1.688	1
(111)	F	2	37	33.114	−3.886	2
(112)	F	4	57	41.291	−15.709	4
(113)	F	3	41	38.438	−2.562	3
(114)	F	3	44	38.076	−5.924	3
(115)	F	2	31	43.038	12.038	4
(116)	F	3	48	45.961	−2.039	1
(117)	F	4	54	44.933	−9.067	3
(118)	F	3	44	50.813	6.813	3
(119)	F	4	63	40.565	−22.435	4
(120)	M	4	60	44.287	−15.713	4
(121)	F	4	59	43.595	−15.405	4
(122)	M	4	51	48.068	−2.932	1
(123)	M	1	28	41.573	13.573	4
(124)	F	1	27	31.497	4.497	2
(125)	F	1	24	31.773	7.773	3

^*∗*^Groupings for the age group considered. ^*∗∗*^Groupings for the age difference between the estimated age and the actual age. ±3 years, group 1; ±5 years, group 2; ±10 years, group 3; >10 years, group 4; subjects from serial numbers 1 to 95 are categorised as “reference group” and 96 to 125 are categorised as “test group.”
